# Supplementary material for: Unique Pakistani gut microbiota highlights population-specific microbiota signatures of type 2 diabetes mellitus
Source: Gut Microbes. 2022 Nov 2;14(1):2142009. doi: 10.1080/19490976.2022.2142009 (PMC9635555; doi:10.1080/19490976.2022.2142009)
Supplement: Supplemental Material [file KGMI_A_2142009_SM0125.zip › Revised Supplemental Material_Supplementary Figures.pdf]

Unique Pakistani gut microbiota highlights population-specific microbiota signatures of type 2 diabetes mellitus

Afshan Saleem[a,b,c], Aamer Ikram[d], Evgenia Dikareva[a], Emilia Lahtinen[a], Dollwin Matharu[a], Anne-Maria Pajari[e], Willem M. de Vos[a,f], Fariha Hasan[b], Anne Salonen[a,*], Ching Jian[a,*,#]

[a]Human Microbiome Research Program, Faculty of Medicine, University of Helsinki, Helsinki, Finland

[b]Department of Microbiology, Faculty of Biological Sciences, Quaid-i-Azam University, Islamabad, Pakistan

[c]Department of Microbiology, Faculty of Basic and Applied Sciences, University of Haripur, Haripur, Pakistan

[d]National Institute of Health, Islamabad, Pakistan

[e]Department of Food and Nutrition, University of Helsinki, Helsinki, Finland

[f]Laboratory of Microbiology, Wageningen University, Wageningen, The Netherlands

[*] Equal contribution

[#]Correspondence:

Ching Jian ching.jian@helsinki.fi

Haartmaninkatu 3, PO box 21, FI-00014, Human Microbiome Research Program, Faculty of Medicine, University of Helsinki, Helsinki, Finland

Figure S1

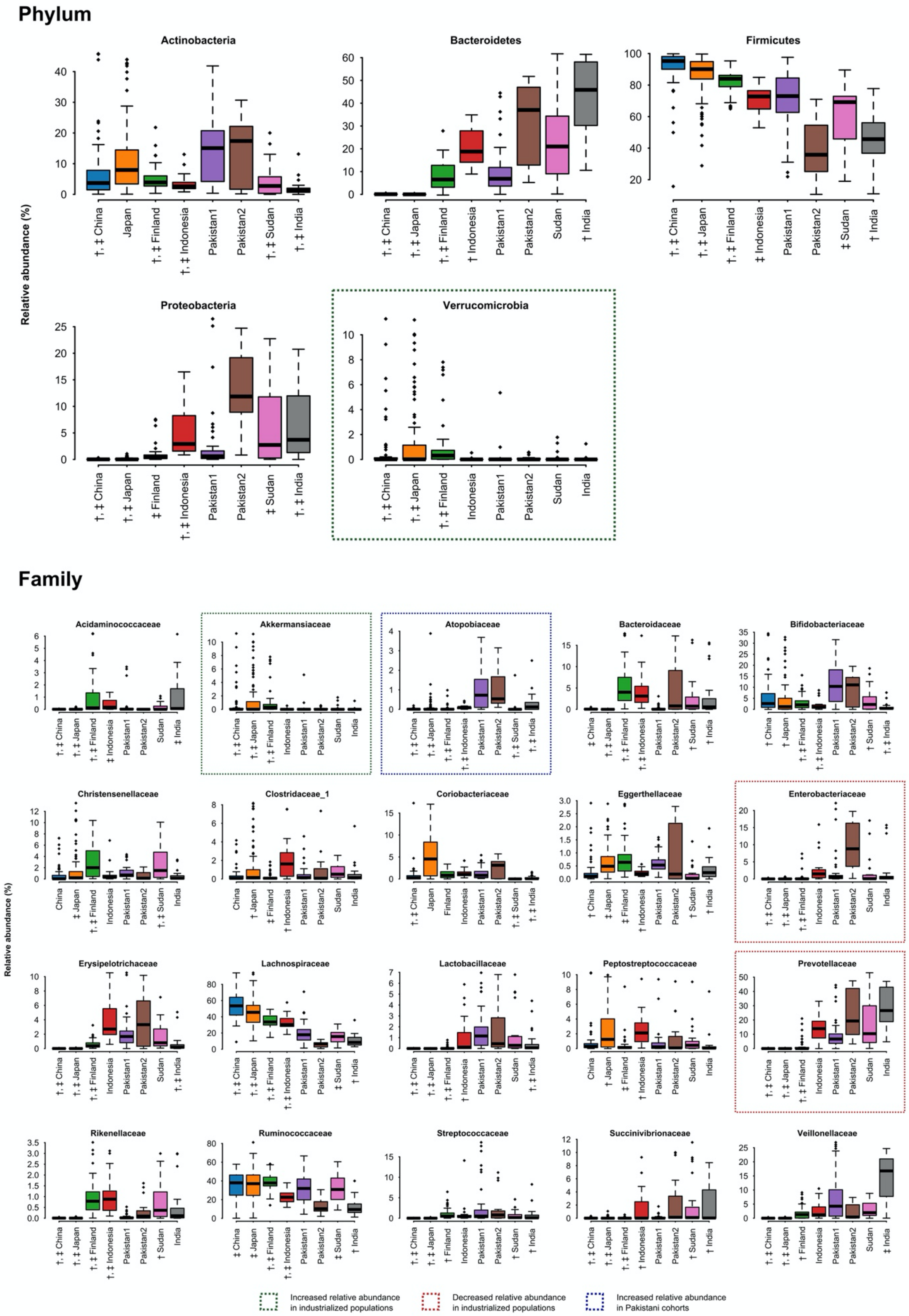
Phylum
Actinobacteria
Bacteroidetes
Firmicutes
Proteobacteria
Verrucomicrobia
Relative abundance (%)
Family
Acidaminococcaceae
Akkermansiaceae
Atopobiaceae
Bacteroidaceae
Bifidobacteriaceae
Christensenellaceae
Clostridaceae_1
Coriobacteriaceae
Eggerthellaceae
Enterobacteriaceae
Erysipelotrichaceae
Lachnospiraceae
Lactobacillaceae
Peptostreptococcaceae
Prevotellaceae
Rikenellaceae
Ruminococcaceae
Streptococcaceae
Succinivibrionaceae
Veillonellaceae
Increased relative abundance in industrialized populations
Decreased relative abundance in industrialized populations
Increased relative abundance in Pakistani cohorts

Figure S1. Box plots depicting the relative abundance of bacterial phyla and top 20 dominant bacterial families per each cohort. *DESeq*2-based differential abundance analysis was performed using Pakistan1 or Pakistan2 as the reference and marked with † or ‡ for statistical significance (FDR-adjusted $P < 0.05$), respectively. The bacterial taxa qualifying as increased or decreased in industrialized populations demonstrated consistent statistical significance in China, Japan and Finland. *Atopobiaceae* was the only bacterial family consistently increased in both Pakistani cohorts in comparison to all other populations.

Figure S2

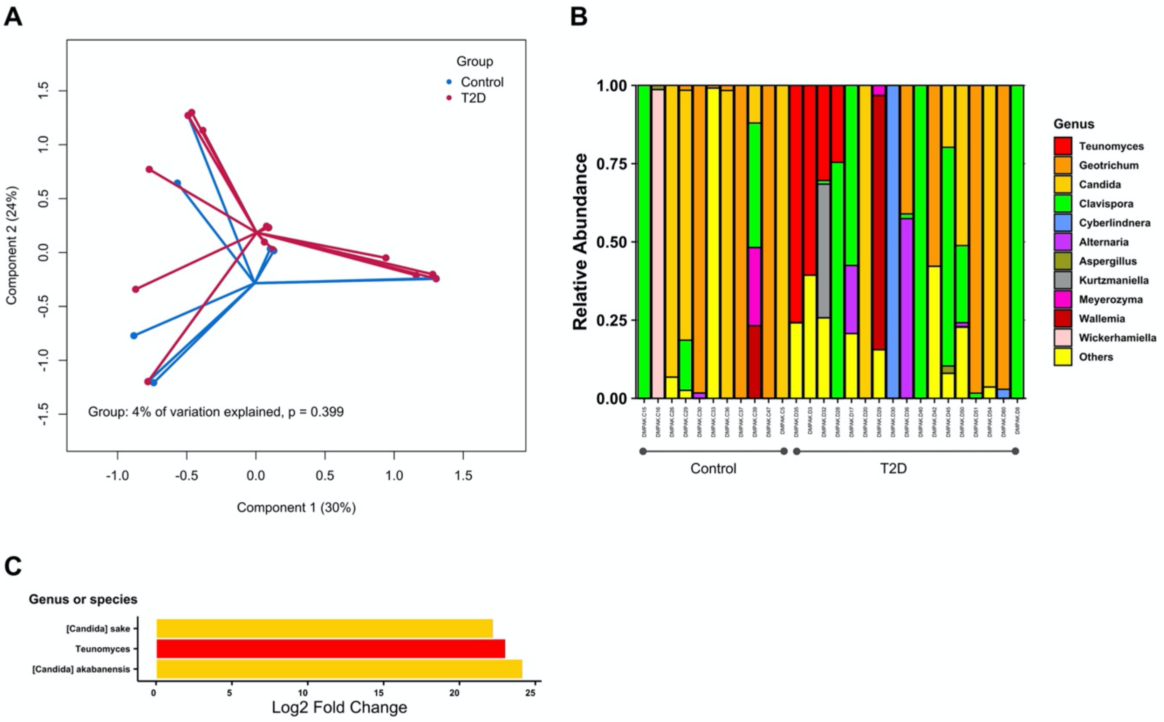

Figure S2. **(A)** Principal coordinate analysis (PCoA) of microbiota variation based on the Bray-Curtis dissimilarity matrix, showing no difference in the overall fungal microbiota between controls (blue) and T2D patients (red) (P = 0.399, PERMANOVA). **(B)** Stacked bar plots showing the composition of fungal genera. **(C)** Differentially abundant fungal genera and species between controls and T2D patients. Only statistically significant results are shown (FDR-adjusted P < 0.05). Log2 fold change was calculated using controls as the reference group.

<u>**Figure S3**</u>

A B

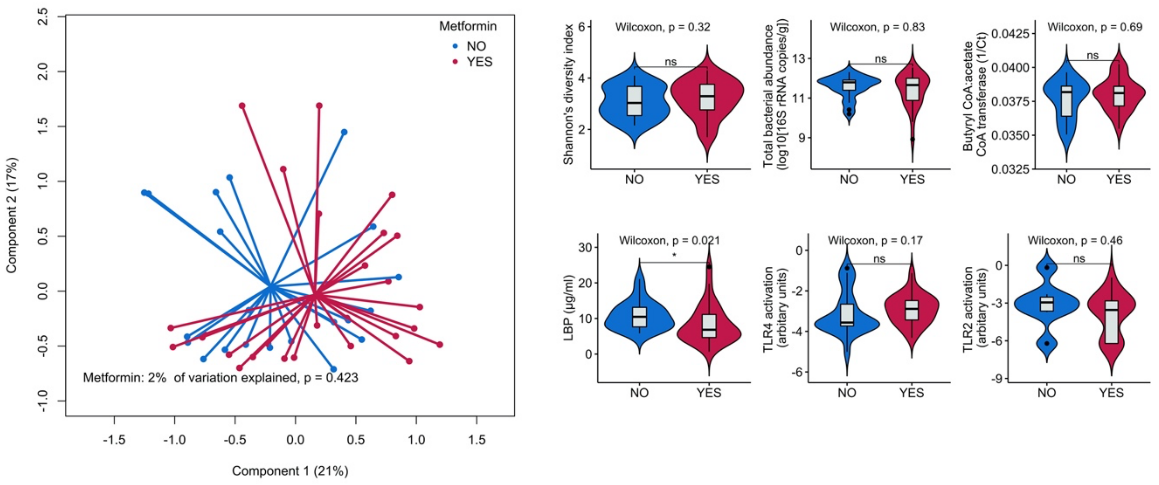

C

Genus	Base Mean	log2 Fold Change	lfcSE	stat	Nominal P	FDR-adjusted P
Family XIII AD3011 group	9.3566	2.0540	0.7556	2.7185	0.0066	0.4813
Bifidobacterium	1100.3311	-0.9930	0.3726	-2.6649	0.0077	0.4813
Ruminiclostridium 6	59.1589	-2.7877	1.0493	-2.6567	0.0079	0.4813
Prevotella 9	9.2291	2.2702	0.9571	2.3721	0.0177	0.8093
Fusobacterium	43.1613	3.1630	1.4137	2.2373	0.0253	0.8409
Negativibacillus	8.1796	2.6013	1.1806	2.2034	0.0276	0.8409

Figure S3. (A) Principal coordinate analysis (PCoA) of microbiota variation based on the Bray-Curtis dissimilarity matrix, showing no statistical difference in the overall gut microbiota between T2D patients treated without (blue) and with (red) metformin (P = 0.399, PERMANOVA **(B)** Comparison of ecological measures of the gut microbiota, butyrate production capacity of the gut microbiota, circulating LBP and TLR4 activation between patients with T2D prescribed with metformin or insulin therapy. * $P < 0.05$; "ns" $P > 0.05$. **(C)** *DESeq*2 outputs showing differentially abundant genera between T2D patients treated without and with metformin that had nominal P values < 0.05. The significant was lost after adjusting for multiple testing (all FDR-adjusted $P > 0.05$). Log2 fold change was calculated using non-metformin users as the reference group.

Figure S4

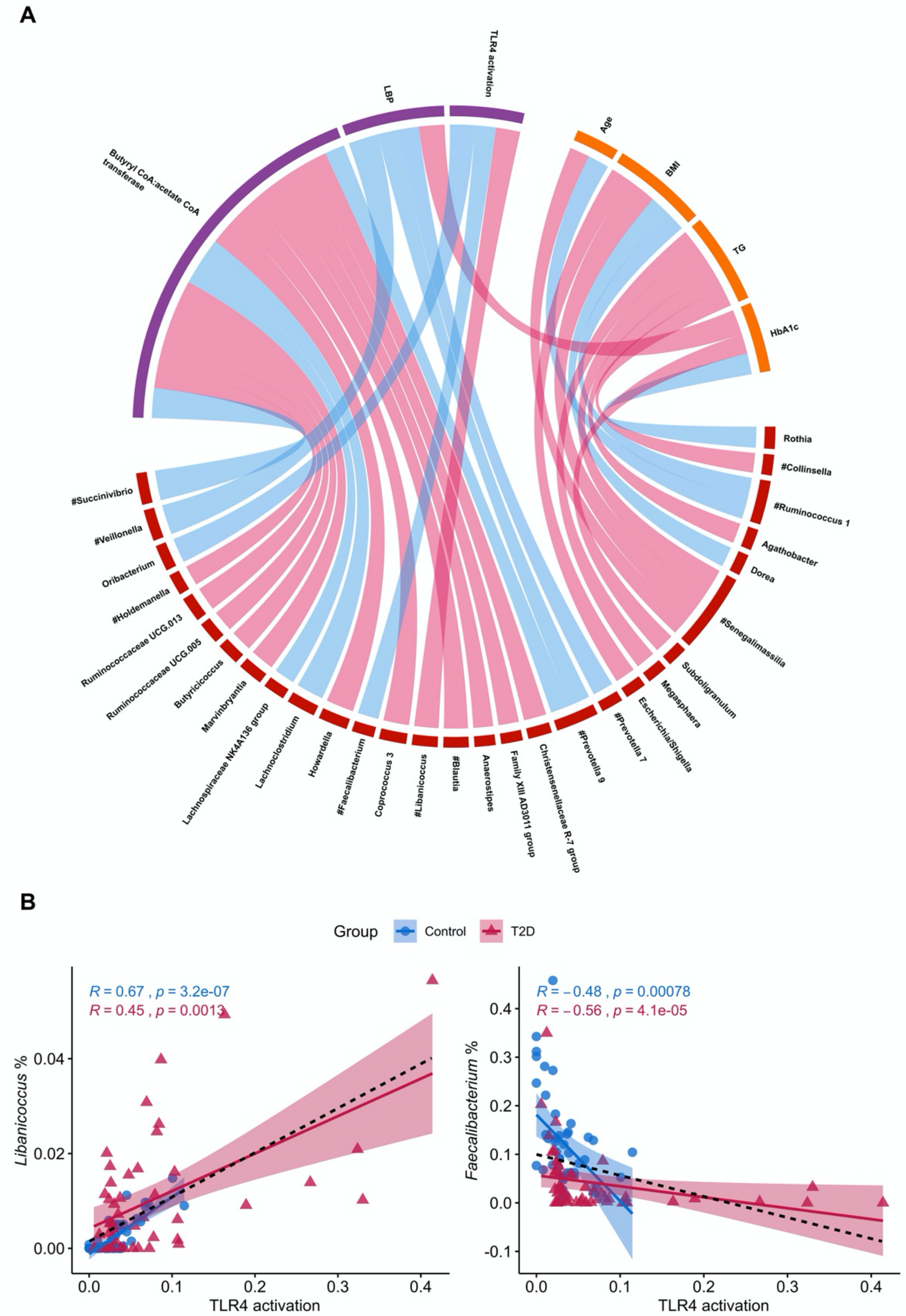

Figure S4. (A) Chord diagram of significant associations in T2D patients as performed in Fig. 6. The bacterial genera consistent with the correlative analysis of the entire cohort are marked with #. **(B)** Subgroup analyses of Spearman's correlation between TLR4 activation and *Faecalibacterium* or *Libanicoccus*. The dashed lines represent the fitted linear regression lines using the entire cohort.

Figure S5

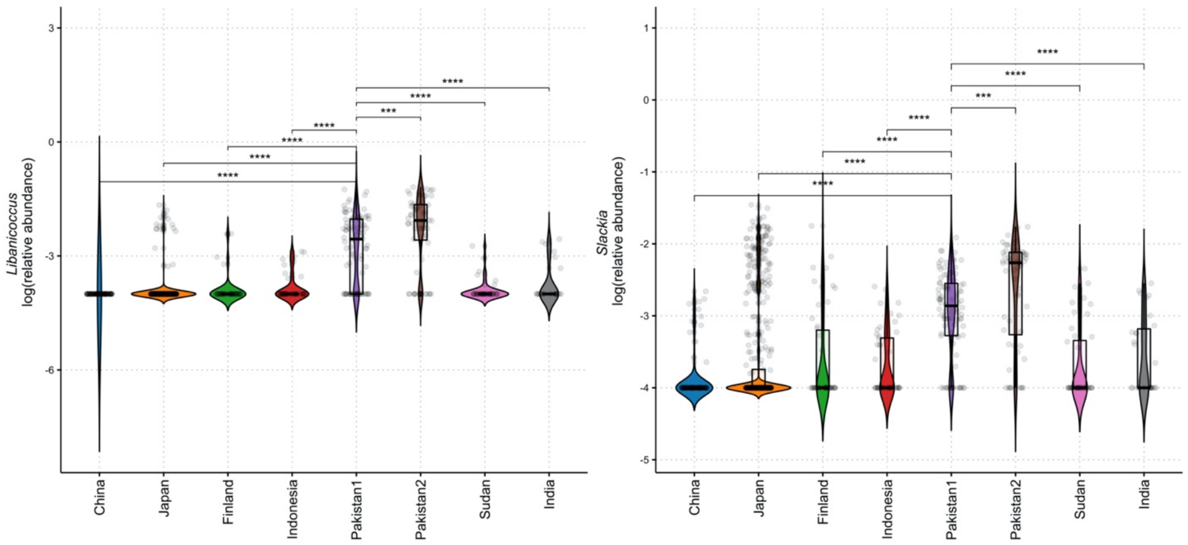

Figure S5. Violin plots (a combination of the box plot with a kernel density plot) showing the relative abundance of *Libanicoccus* and *Slackia* per each cohort. The center line denotes the median, the boxes cover the 25th and 75th percentiles, and the whiskers extend to the most extreme data point, which is no more than 1.5 times the length of the box away from the box. Points outside the whiskers represent outlier samples. Significance was calculated by the Wilcoxon rank-sum test using Pakistan1 as the reference. **** P < 0.0001; *** P < 0.001; ** P < 0.01; * P < 0.05.

Figure S6

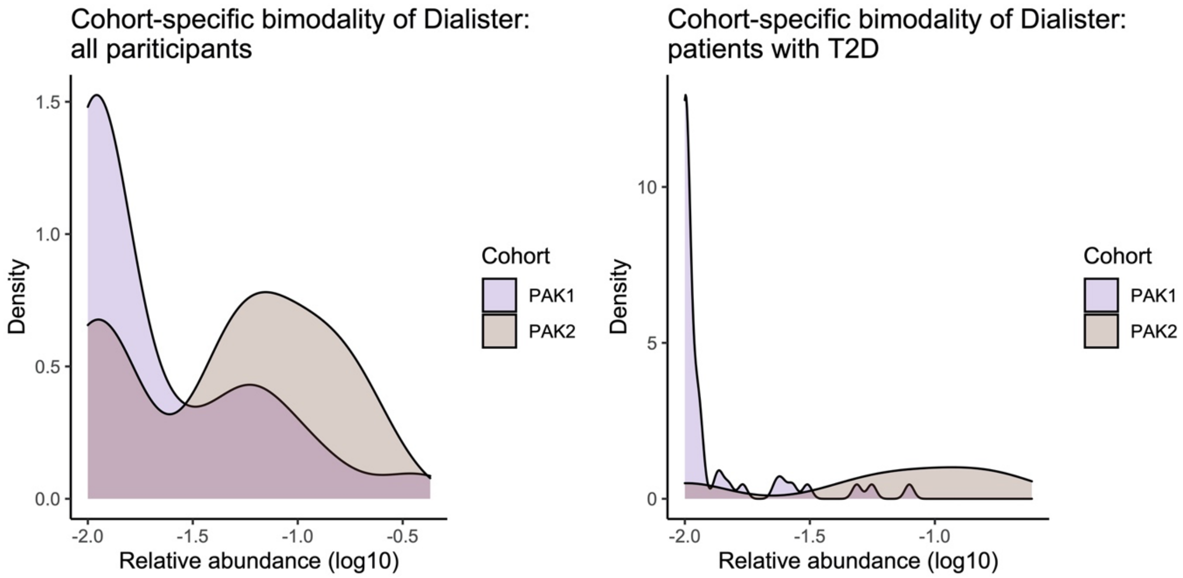

Figure S6. Observation density of *Dialister* in controls (left) and T2D patients (right).

Figure S7

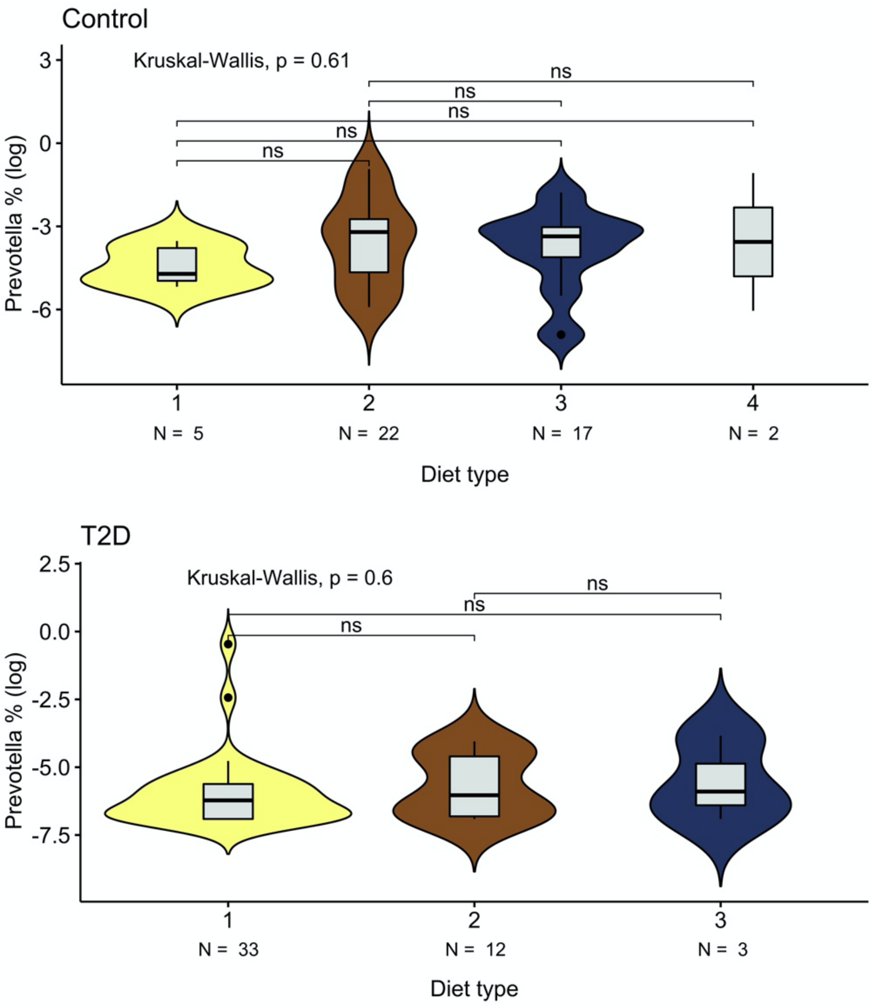

Figure S7. Violin plots (a combination of the box plot with a kernel density plot) showing relative abundance of *Prevotella* according to diet (Table S5). The center line denotes the median, the boxes cover the 25th and 75th percentiles, and the whiskers extend to the most extreme data point, which is no more than 1.5 times the length of the box away from the box. Points outside the whiskers represent outlier samples. Significance was calculated using the Wilcoxon rank-sum test. "ns", $P > 0.05$.
